# Accelerating Electrolyte Discovery Using Generative Models and Molecular Dynamics Simulations

**DOI:** 10.1021/acsomega.6c01811

**Published:** 2026-08-10

**Authors:** Varisara Deerattrakul, Salatan Duangdangchote, Adisak Boonchun, Pawin Iamprasertkun

**Affiliations:** † School of Bio-Chemical Engineering and Technology, Sirindhron International Institute of Technology, 37698 Thammasat University, Pathum Thani 12120, Thailand; ‡ Research Unit in Sustainable Electrochemical Intelligent, Thammasat University, Pathum Thani 12120, Thailand; § Maple Synapse Inc, North York, Ontario M2K 0C4, Canada; ∥ Department of Physics, Faculty of Science, 54775 Kasetsart University, Bangkok 10900, Thailand

## Abstract

Operating lithium-ion batteries (LIBs) at high voltages and low temperatures demands electrolyte solvents with highly tailored electronic properties. Because traditional discovery is limited by historical solvent libraries and human intuition, expanding the accessible chemical space requires advanced computational methods. Here, we deploy the generative machine learning model G-SchNet, trained on the QM9-GCDQE database, to invert the design process for three designed targeted electrolyte environments: general-purpose, high-voltage, and low-temperature. We filtered the generated molecular space using a multistage framework based on validity, uniqueness, and novelty, followed by DFT and molecular dynamics (MD) validation. We identified a computationally promising candidate, trifluoro­((fluoromethoxy)­methoxy)­methane, which is a fluorinated ether that exhibits computed transport properties enhanced relative to a conventional EC/DEC electrolyte under identical simulation conditions. This workflow illustrates the capacity of generative models to identify computationally promising electrolyte candidates that warrant further computational and experimental investigation.

## Graphical abstract

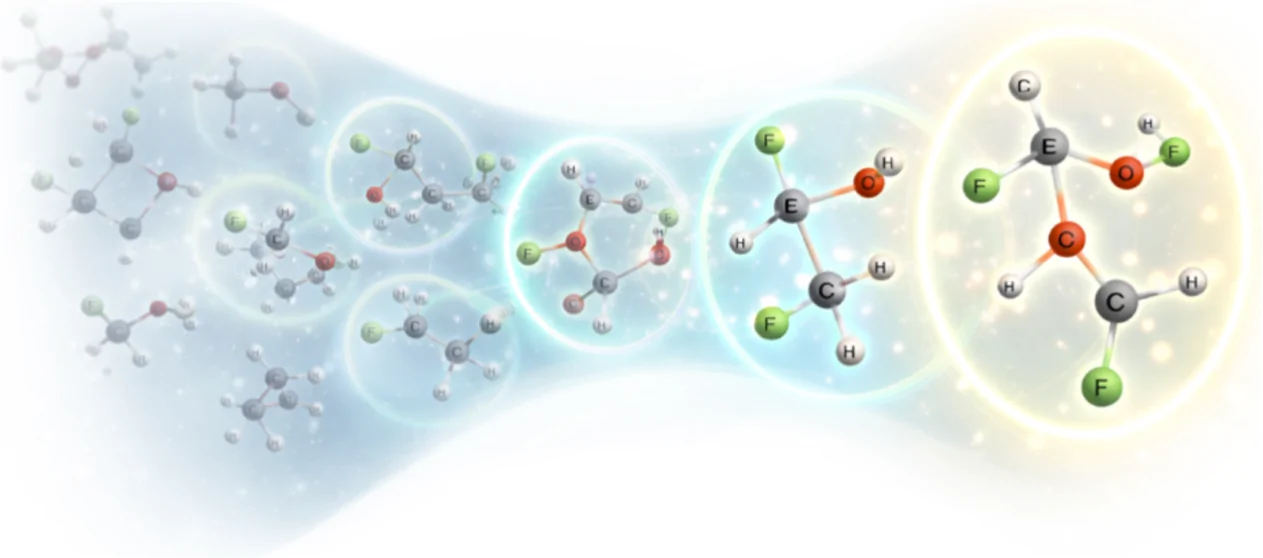

## Introduction

The increasing demands on for global energy storage, driven by electric vehicles and grid-scale storage, continue to raise the performance bar for (LIBs).^1,2^ While electrode materials have seen steady improvements in energy density, the electrolyte remains a critical bottleneck, particularly for operations requiring high voltage stability (>4.5 V) and low-temperature conductivity.^3–5^ Commercial electrolytes, predominantly composed of cyclic and linear carbonates such as ethylene carbonate (EC) and diethyl carbonate (DEC), suffer from narrow electrochemical stability windows and sluggish ion transport at subzero temperatures. Although the industry has historically relied on additives like fluoroethylene carbonate (FEC) to stabilize the solid–electrolyte interphase (SEI),^6^ these solutions often impose a penalty in viscosity that compromises high-rate capability. Consequently, the discovery of novel solvent architectures that intrinsically balance wide electrochemical windows with rapid ion transport remains an important goal for modern battery chemistry.^7–10^

Traditional electrolyte discovery has long been dominated by chemical intuition and iterative experimental screening. While effective for incremental improvements, the trial-and-error approach is inefficient for exploring the vast chemical space of potential organic solvents, which is estimated to exceed 10^60^ distinct molecules.^11,12^ To overcome these limitations, recent research has pivoted toward data-driven methodologies. High-throughput computational screening, often coupled with density functional theory (DFT), has been employed to evaluate large libraries of known compounds for properties such as redox potential and solvation energy.^13^ More recently, machine learning (ML) has emerged as a powerful tool to accelerate this process.^14–17^

However, most of these existing computational approaches are selective filtering rather than generative; they are limited to screening known libraries or optimizing existing formulations. To explore chemical spaces beyond known databases, an inverse design paradigm using generative deep learning.^18^ Generative models, such as variational autoencoders (VAEs)^19^ and generative adversarial networks (GANs),^20^ have shown promise in drug discovery, but their application remains underexplored in electrolyte discovery for energy storage applications. In this work, we employ G-SchNet^21^ with SchNetPack 2.0,^22^ a continuous-filter convolutional neural network designed for generating 3D molecular structures. We selected this specific architecture because, unlike string-based models (e.g., SMILES generators), G-SchNet explicitly captures the spatial symmetries and rotational invariances of molecules, which are critical for accurately predicting the quantum mechanical descriptors, HOMO, LUMO, and dipole moment, that are foundational descriptors for initial electrolyte screening. We also include vertical ionization potential, electron affinity,^23^ and a synthetic-accessibility score^24^ during the screening state which together provide a more insights into molecular and electronic properties in order to comparison with the conventional electrolyte solvents.

As a proof of concept, we combine this generative capability with high-throughput molecular dynamics (MD) simulations to establish a sequential generation, screening, and validation pipeline. While generative models can propose chemically valid structures, they cannot predict bulk transport phenomena such as viscosity or ionic conductivity. Therefore, we utilize MD simulations with the OPLS-AA force field to validate the dynamic behavior of the AI-generated compounds, employing transport quantities (diffusion coefficient, Nernst–Einstein conductivity, and transference number). This multiscale approach allows us to not only identify molecules with the electronic signatures for high-voltage stability but also ensure they possess the necessary solvation dynamics for rapid lithium transport. Through this pipeline, we identify a class of fluorinated ether solvents that show computationally promising transport properties relative to conventional carbonate solvents, providing initial targets that warrant experimental investigation.

## Experimental Section

### Generative Model Architecture and Training

The G-SchNet^21^ model was used to produce 3D molecular structures conditioned on target electronic properties. At each generation step, the model predicts (i) the element type of the next atom and (ii) a probability distribution over distances to all previously placed atoms, from which the 3D position of the new atom is sampled. The model was conditioned on three simultaneous target properties, including HOMO energy (range: −12.0 to −2.0 eV), LUMO energy (range: −7.0 to 5.0 eV), and dipole moment (range: 0.0 to 15.0 D), via a dedicated conditioning module. The model was trained on the QM9-GCDQE data set,^25^ containing 443,106 molecules composed of hydrogen, carbon, nitrogen, oxygen, and fluorine. The data set was split in a ratio of 70:20:10 for training, validation, and testing, respectively. Training was performed using the AdamW optimizer (learning rate = 1 × 10^–4^) with a batch size of 100. The learning rate was reduced on plateau (factor = 0.5, patience = 10 epochs, cooldown = 10 epochs, minimum LR = 1 × 10^–6^). Early stopping with a patience of 25 epochs on the validation loss was employed. Additional training details are provided in Supporting Information.

### Target Profiles and Generation

We defined three distinct target profiles to direct the generation process. Group I (General) targeted a balanced electronic profile (HOMO ∼ −7.2 eV, Dipole ∼ 4.0 D).^4,26^ Group II (High Voltage) targeted deep HOMO levels (<−8.4 eV) to promote resistance against oxidative decomposition at high potentials, paired with a high dipole moment (5.5 D) to maintain salt solubility.^27–29^ Group III (Low Temperature) prioritized moderate polarity (Dipole ∼ 2.2 D) to minimize the desolvation energy, which is often the rate-limiting step at low temperatures.^30–33^ For each group, 5000 molecules were generated using the trained model with ancestral sampling.

### Chemical Validity and Screening

Generated 3D structures were processed through a multistage automated screening pipeline. First, a 3D-to-SMILES conversion was performed to simultaneously verify structural integrity: bond connectivity was inferred from atomic positions, and the resulting molecular graphs were converted to canonical SMILES using RDKit.^34^ Molecules were classified as valid only if they (i) produced a parseable SMILES representation of a neutral (charge = 0) species and (ii) contained no radical electrons. This step implicitly enforces correct valence, elemental consistency (H, C, N, O, F only), and chemically reasonable 3D geometries; structures with unphysical bond lengths, incorrect atom connectivity, or strained arrangements that cannot be resolved into a valid Lewis structure are rejected at this stage. Second, canonical SMILES strings were compared to identify and remove duplicate structures (grouped by molecular formula, with stereoisomers treated as distinct). Third, the remaining unique molecules were compared against the QM9-GCDQE training and validation databases to identify novel structures that do not present in the training data. Quantitative validation of the generative model including validity rate, uniqueness rate, novelty rate, conditioning fidelity (mean absolute error between target and DFT-computed property values), and Tanimoto similarity to the nearest training-set neighbor is reported in Table S2 and Figure S8.

### DFT Calculations

Electronic properties of generated molecules and the QM9-GCDQE training data set (HOMO, LUMO, and dipole moment) were computed at the B3LYP/6-31G­(d) level of theory with geometry optimization and frequency analysis using Gaussian 16.^35^ For candidates A–F and reference solvents (EC, DEC, DMC, FEC), we additionally computed: vertical ionization potential (vIP) and vertical electron affinity (vEA) from single-point energies of the cation- and anion-radical states at the optimized neutral geometry. The synthetic-accessibility SAscore^24^ was computed from canonical SMILES. These descriptors are reported in the Table 1.

**Table 1:** Electronic and Physicochemical Properties of Conventional Electrolyte Solvents and Generated Molecules

solvent	HOMO (eV)	LUMO (eV)	μ (D)	vIP (eV)	vEA (eV)	SAscore	source
EC	–8.23	0.626	5.3	8.07	–0.79	2.79	^26,54^
DEC	–7.88	1.03	1.1	7.86	–1.14	1.81	^26,54^
DMC	–7.98	0.989	0.3	8.17	–1.17	2.26	^26,54^
FEC	–8.70	0.309	5.6	8.34	–0.53	4.11	^26,54^
cand. A	–6.83	2.26	1.78	6.59	–2.08	2.79	this work
cand. B	–6.90	0.37	2.56	6.36	–0.20	4.31	this work
cand. C	–7.75	–0.48	2.69	7.00	0.43	4.68	this work
cand. D	–8.74	2.45	0.75	7.83	–2.18	3.42	this work
cand. E	–6.54	0.45	2.19	6.15	–0.03	4.52	this work
cand. F	–6.23	0.50	1.07	6.15	–0.03	4.52	this work

### MD Simulation Protocol

The classical molecular dynamics (MD) simulations were performed using the optimized potentials for liquid simulations for all-atom (OPLS-AA)^36–39^ force field for bonded interactions (bonds, angles, dihedrals, and impropers). The partial atomic charges for all molecules were obtained by first optimizing the geometry using B3LYP^40^ level of theory based on density functional theory (DFT) with the aug-cc-pvdz basis set using the Gaussian 16 simulation package.^35^ Subsequently, the electrostatic potential surface was fitted by the RESP^41,42^ method. An approximately 55 Å cubic simulation boxes were constructed with randomly placed 70 Li^+^, 70 PF_6_^–^, and 600 total solvent molecules using PACKMOL^43^ (∼1 M). The initial configuration was then energy minimized with a conjugate gradient algorithm for 20,000 steps. Following the minimization, the system was equilibrated in the isothermal–isobaric (*NPT*) ensemble for 2 ns with a time step of 0.5 fs at a temperature of 300 K. For production runs, 20 ns simulations were carried out in the *NVT* ensemble with the Nosé–Hoover chain (NHC)^44^ thermostat, a time step of 1 fs, and a temperature of 300 K. The last 4 ns of the production runs were used for the MD analysis in this publication. All classical MD simulations were performed with the GROMACS^45–50^ simulation package on a 24-core Unix-based cluster. Self-diffusion coefficients D were extracted from the Einstein relation in the linear regime of the mean square displacement (MSD). The Li^+^ transference number *t*_Li^+^_ was computed from the ratio of ionic diffusivities. Ionic conductivity σ was estimated using the Nernst–Einstein relation. The static dielectric constant ε was estimated from total-dipole fluctuations of the simulation cell. We note that the nonpolarizable OPLS-AA force field systematically underestimates absolute Li^+^ diffusion by 1–2 orders of magnitude; our conclusions therefore rest on relative rankings between systems simulated under identical protocols, and absolute quantitative predictions should be interpreted with appropriate caution.

## Results and Discussion

### Training Data Set Characterization

The training data set provides a chemically diverse scaffold dominated by small organic molecules with an average of 20 atoms, making a reasonable starting point for solvent design (Figure 1). The elemental composition is heavily weighted toward carbon and hydrogen, with oxygen, nitrogen, and fluorine present in varying proportions. The HOMO distribution spans approximately −11 to −5 eV, the LUMO distribution spans −4 to +6 eV, and the dipole moment distribution ranges from 0 to ∼ 10 D, providing broad coverage of the target property space for electrolyte design.

**Figure 1:**
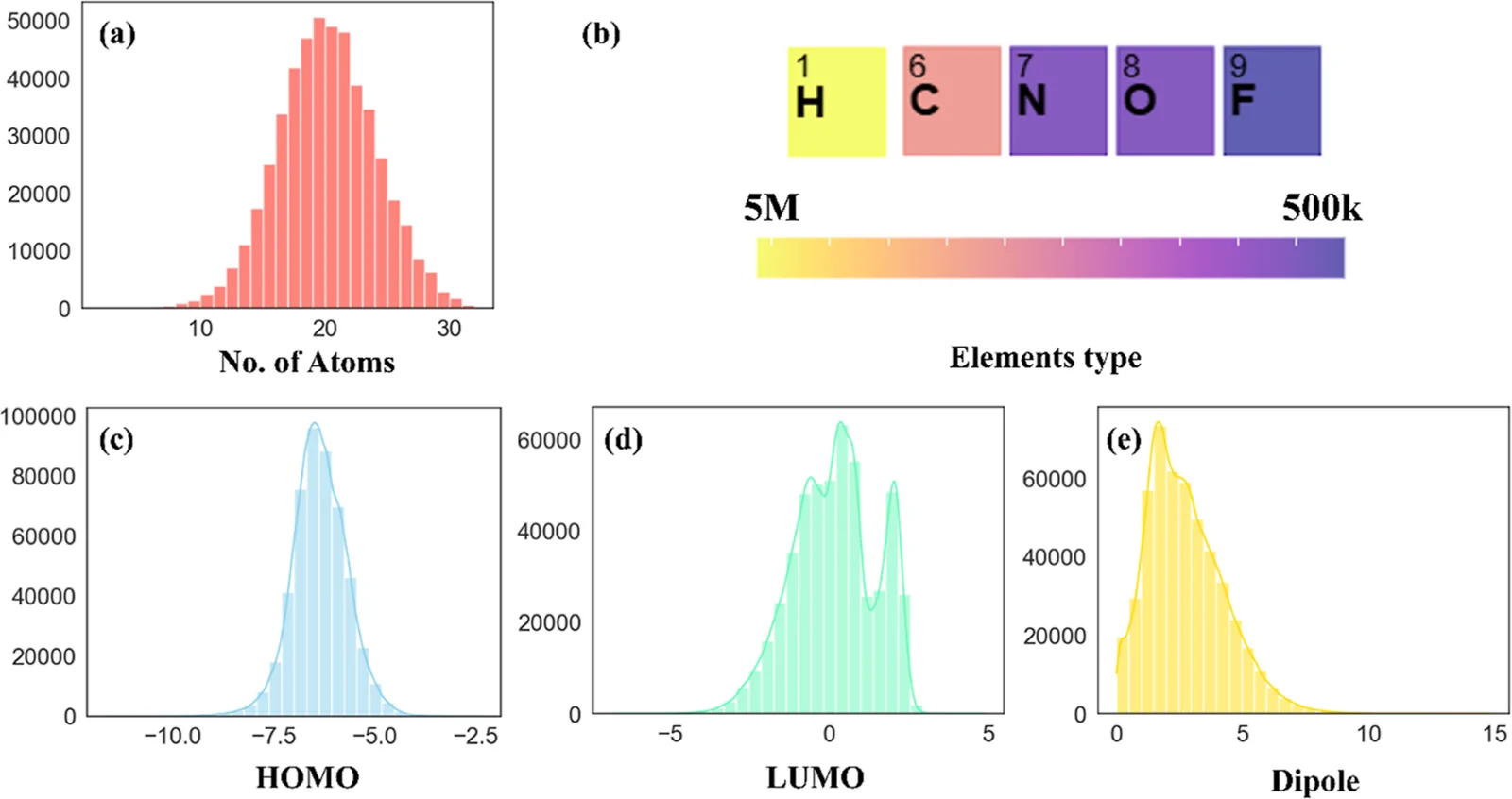
Characterization of the QM9-QCDGE training library showing (a) atom count distribution, (b) elemental composition, and (c–e) the distribution of HOMO, LUMO, and dipole moment values.

### Generative Model Validation

The generative model exhibited good fidelity in reproducing the targeted electronic properties across all three groups. The distribution of generated HOMO and LUMO values aligned closely with the specified targets, demonstrating the model’s ability to learn complex structure–property relationships (Figure 2). The training and validation loss curves (Figure S2) confirm smooth convergence with minimal overfitting, and the test set loss is consistent with the validation loss, indicating robust generalization. Quantitative validation metrics are reported in Table S2: the model achieves a validity rate of more than 70% across all three target groups, whereas all valid structure produced a parseable, neutral, and radical-free. All structure groups have a uniqueness rate of 100.0%, and a novelty rate of 91.1%, 90.1%, and 94.7% for Groups I, II, and III, respectively, against the QM9-GCDQE training and validation sets. Conditioning fidelity, measured as the mean absolute error between target and DFT-computed property values, is 0.47–0.83 eV for HOMO, 1.88–2.76 eV for LUMO, and 1.05–2.03 D for dipole moment across the three groups. The tighter HOMO conditioning relative to LUMO is consistent with the well-known asymmetry in the predictability of frontier orbitals: occupied orbitals are anchored to filled bonding states, whereas virtual orbitals are more sensitive to basis-set and exchange–correlation choices.^51^ Structural novelty was further assessed by computing the Tanimoto similarity^52^ of each generated molecule to its nearest neighbor in the training set: the median similarity is 0.536, with 36.9% of generated molecules having similarity below 0.5, a commonly used threshold for chemically distinct substructure (Figure S8). The combination of structural uniqueness, novelty against training, and a moderate median fingerprint similarity is consistent with a generative model that explores nearby chemical space within the QM9-GCDQE elemental envelope, proposing new combinations of familiar substructures rather than entirely new molecular scaffolds. This is the expected and appropriate behavior for an inverse-design model trained on a constrained data set and supports the interpretation that the model produces designed chemistry rather than memorized training molecules. However, a divergence was observed in the dipole moment distribution for groups II and III, where the generated molecules consistently exhibited dipole moments slightly lower than the targeted 5.5 D value. This observation likely arises from a physicochemical constraint: the incorporation of highly electronegative fluorinated groups, necessary for lowering the HOMO to achieve high-voltage stability, often introduces opposing polarity vectors that reduce the net molecular dipole.^53^

**Figure 2:**
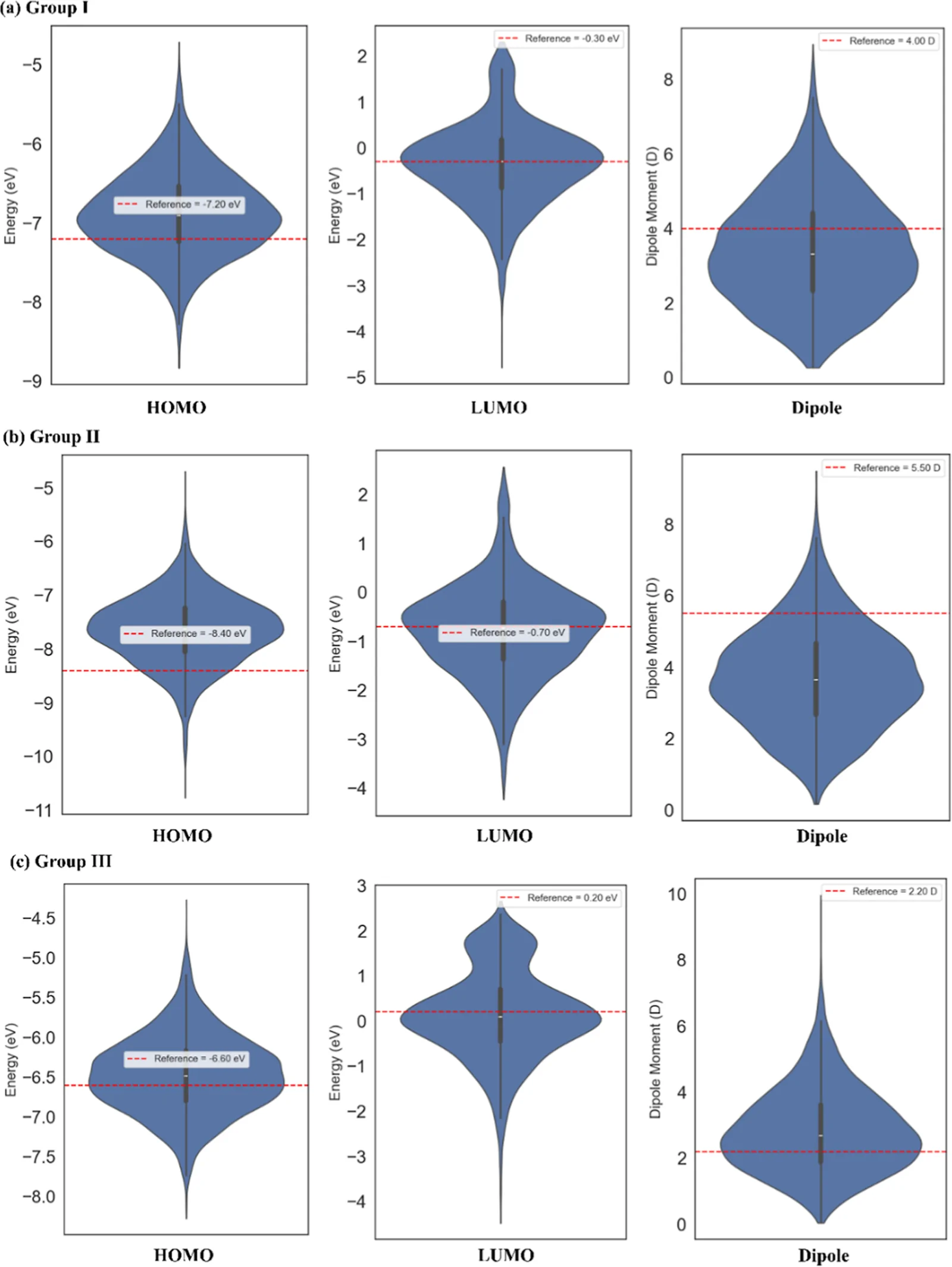
Statistical validation of the generative model. Violin plots for (a) group I, (b) group II, and (c) roup III. Red dashed lines indicate target reference values.

### Candidate Selection and Characterization

Despite this constraint, the screening pipeline yielded a diverse array of stable candidates, predominantly featuring ether, ketone, and nitrile functionalities (Figure S3). The chemical structures of the six selected candidates are shown in Figure 3. For detailed electronic and transport characterization, we selected six candidates, labeled A through F, which chosen to span the functional-motif diversity present in the top-ranked output of the composite ranking: fluorinated ethers (A and D), substituted dihydropyrans (B and C), a methylenetetrahydropyran (E), and a bicyclic dioxin–dioxolane (F). Candidates A and D are notable as fluorinated ethers, specifically identified as 1-(fluoromethoxy)-2-methoxyethane (A) and trifluoro­((fluoromethoxy)­methoxy)­methane (D). These structures share substantial homology with hydrofluoroethers (HFEs), a class of solvents valued for their nonflammability and low viscosity, and they feature oxygen coordination sites that may facilitate lithium solvation.

**Figure 3:**
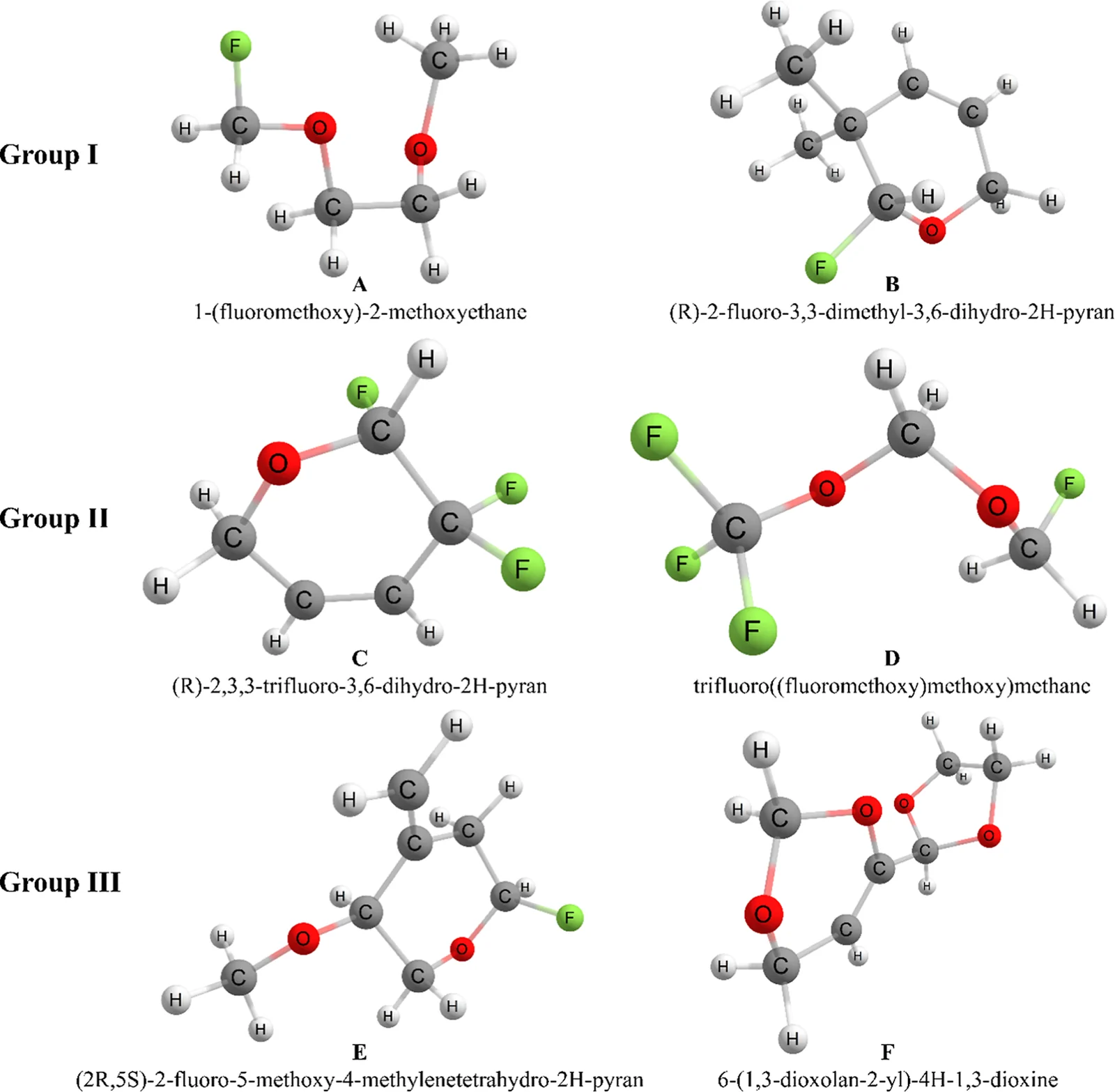
Chemical structures of candidates electrolyte molecules (A) 1-(fluoromethoxy)-2-metgoxyethane, (B) (R)-2-fluoro-3,3-dimethyl-3,6-dihydro-2*H*-pyran, (C) (R)-2,3,3-trifluoro-3,6-dihydro-2*H*-pyran, (D) trifluoro­((fluoromethoxy)­methoxy)­methane, (E) (2*R*,5*S*)-2-fluoro-5-methoxy-4-methylenetetrahydro-2*H*-pyran, and (F) 6-(1,3,-dioxolan-2-yl)-4*H*-1,3-dioxine.

### Benchmarking Against Conventional Solvents

To place the generated candidates in appropriate scientific context, Table 1 presents a systematic comparison of the electronic and physicochemical properties of candidates A–F against five widely used electrolyte solvents: ethylene carbonate (EC), diethyl carbonate (DEC), dimethyl carbonate (DMC), and fluoroethylene carbonate (FEC). Computed DFT values, including vertical ionization potential (vIP), vertical electron affinity (vEA), and synthetic accessibility (SAscore) are presented alongside experimental literature values where available.

The comparison shows that candidate D exhibits the deepest HOMO among the candidates (−8.74 eV), deeper than EC (−8.23 eV) and DEC (−7.88 eV), and FEC (−8.70 eV). However, the vertical ionization potential, places candidate D at 7.83 eV, marginally below EC (8.07 eV), DEC (7.86 eV), and FEC (8.34 eV). The deep HOMO of candidate D does not translate into superior oxidative stability when the full ionization cost is computed. Candidate D is therefore best characterized as offering oxidative stability comparable to conventional carbonate electrolytes. At the same time, candidate D shows a notably negative vertical electron affinity (vEA = −2.18 eV), substantially more negative than EC (−0.79 eV), indicating strong resistance to reduction. Across the descriptor set, candidate D thus presents a balanced electrochemical profile rather than a high-voltage breakthrough: oxidative stability matching the carbonate baseline and reductive stability superior to EC. Candidate C also shows a relatively deep HOMO (−7.75 eV), comparable to DMC (−7.98 eV). In contrast, candidates A, B, E, and F exhibit shallower HOMO levels (−6.2 to −6.9 eV), indicating that their value lies not in oxidative resistance but in other properties such as low molecular weight (A, 108 g/mol), moderate polarity (B, E), or structural novelty (F, the only fluorine-free candidate). Candidate D’s very low dipole moment (0.75 D) suggests weak Li^+^ coordination, which is consistent with the contact ion pair formation and enhanced transport observed in the MD simulations. This weak-solvation design philosophy mirrors recent developments in fluorinated ether cosolvents for high-voltage and localized high-concentration electrolytes.^55–57^ Synthetic accessibility scores (SAscore) for the candidates range from 2.79 (A) to 4.68 (C), comparable to the most synthetically demanding reference (FEC, SAscore 4.11) but higher than the simpler carbonates (DEC 1.81, DMC 2.26, EC 2.79). Candidate D’s SAscore of 3.42 indicates synthetic accessibility comparable to FEC, suggesting practical synthetic routes are not the primary barrier to experimental evaluation.

### Transport Properties

The bulk transport properties derived from the MD simulations provide an initial computational assessment of the candidates’ potential as electrolyte solvents. The MSD trajectories reveal a notable separation in diffusive behavior between the generated candidates and the conventional control (Figure S5). Among the candidates examined, candidate D exhibited the highest computed Li^+^ diffusion coefficient (*D*_Li^+^_) of 15.09 ± 5.50 × 10^–11^ m^2^ s^–1^, compared to 0.48 ± 0.13 × 10^–11^ m^2^ s^–1^ for the EC/DEC control under identical simulation conditions. While the magnitude of this difference is notable, we caution that absolute diffusion values from classical MD with nonpolarizable force fields should be interpreted with care, particularly for fluorinated solvents (see Methods, Methodological Limitations). The relative ranking among candidates, however, is expected to be more reliable. Candidate A also showed enhanced transport (13.93 ± 9.56 × 10^–11^ m^2^ s^–1^). In contrast, candidates C and E exhibited diffusion coefficients only marginally higher than the control, despite satisfying the target electronic criteria. This observation suggests that electronic descriptors alone are insufficient to predict bulk transport, and that molecular shape, flexibility, and solvation shell geometry play important mediating roles, a finding that motivates the incorporation of additional descriptors in future iterations of the workflow.

The Li^+^ transference number (*t*_Li^+^_) for candidate D was calculated to be 0.50, compared to 0.35 for the EC/DEC (Figure 4). If confirmed experimentally, a higher transference number would help minimize concentration polarization during high-rate charging, suggesting that candidate D could potentially offer advantages in power density applications.

**Figure 4:**
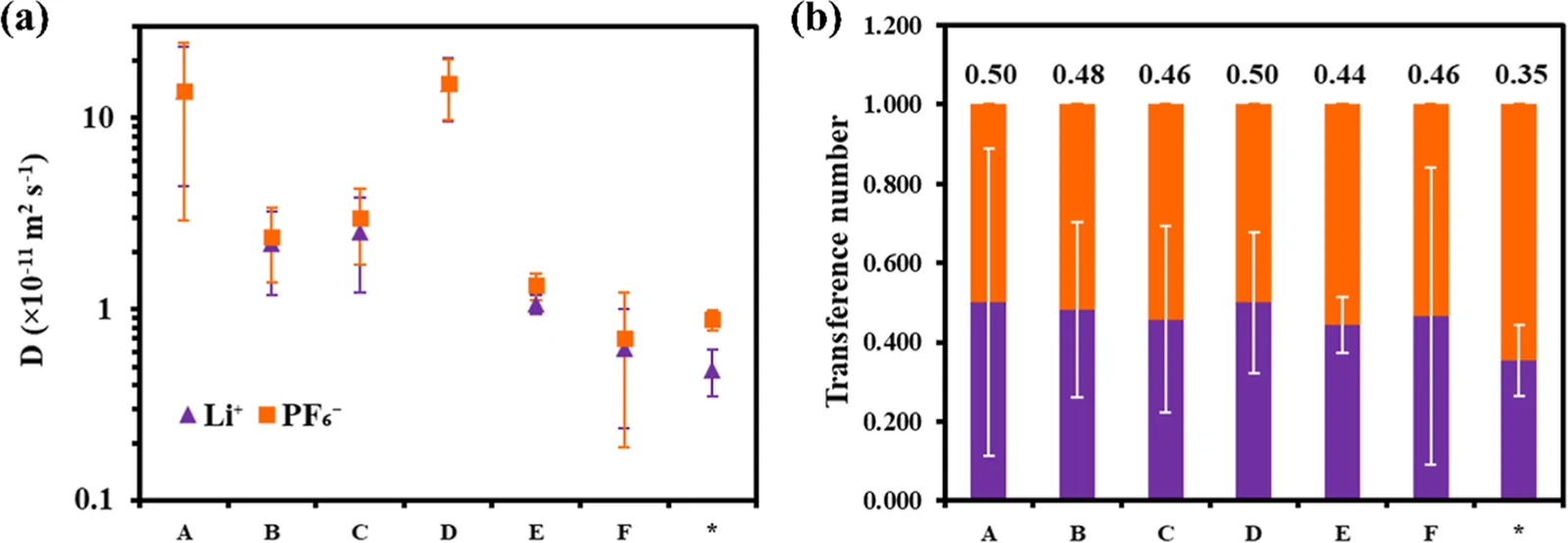
Comparative transport properties. (a) Self-diffusion coefficients (*D*), and (b) transference numbers.

### Solvation Structure Analysis

To explore the atomistic origins of this enhanced transport, we analyzed the radial pair distribution functions (RDFs) and their corresponding coordination number integrals (Figures S6 and S7). In the control electrolyte, the solvation shell is dominated by the carbonyl oxygens of EC, forming a tight coordination environment that largely separates Li^+^ from the PF_6_^–^ anion. Conversely, the RDFs for candidates A and D suggest a different solvation structure where the fluorinated ether oxygens coordinate with lithium in a geometry that permits a higher degree of anion interaction.

The coordination number analysis indicates that candidates A and D appear to favor the formation of contact ion pairs (CIPs) within the first solvation shell. While conventional electrolyte design often seeks to maximize ion dissociation, recent literature suggests that excessive dissociation can lead to high viscosity and high desolvation energy barriers.^58–60^ The fluorinated ethers identified here may occupy an intermediate regime: they interact weakly enough with Li^+^ to enable low desolvation penalties, while their low viscosity facilitates collective transport of the ion–solvent cluster.^60,61^ This interpretation is broadly consistent with recent findings in high-concentration electrolyte research, where weak-binding solvents have been shown to enhance overall conductivity by modifying the residence time of the solvation sheath.^58,62^

## Conclusion

This study presents a proof-of-concept computational workflow for the design of battery electrolytes. By leveraging the spatial awareness of the G-SchNet generative model, we explored a chemical space of over 400,000 molecules to identify fluorinated ether candidates that show computationally promising properties relative to commercial carbonate electrolytes. The generative model produced chemically valid, structurally unique, and largely novel candidates (∼74% structural validity rate against 5000 generations per group, 100% uniqueness within validated pools, and ∼ 92% novelty against the training set), with HOMO conditioning fidelity of 0.47–0.83 eV. Our lead candidate, trifluoro­((fluoromethoxy)­methoxy)­methane (candidate D), exhibits electronic stability descriptors comparable to conventional carbonates (vIP 7.83 eV) with notably stronger resistance to reduction (vEA −2.18 eV), and computed transport properties that are enhanced relative to the EC/DEC. The MD simulations also suggest that this transport behavior may be related to a solvation mechanism that balances weak solvent coordination with controlled ion pairing. Candidate D thus emerges as a computationally promising screening hit, with oxidative stability comparable to conventional carbonates and notably enhanced reductive stability, which should warrant further computational and experimental evaluation.

Several important limitations must be noted. The generative model is constrained to the elemental palette and molecular size range of the QM9-GCDQE training set. The design criteria (HOMO, LUMO, dipole moment) represent necessary but might be not sufficient conditions for real-world electrolyte performance; properties such as SEI-forming tendency, flammability, chemical stability toward electrode materials, and long-term cycling behavior which require further computational and/or experimental investigations, especially for low-temperature applications. The MD simulations, while providing internally consistent relative rankings, are subject to force field approximations that systematically underestimate absolute transport values. Moreover, to ensure valid comparisons, one should be aware that all analyses must be performed under identical, force-field-dependent simulation conditions. The six candidates examined represent a small subset of the generated chemical space.

Despite these limitations, this work demonstrates that generative deep learning, combined with physics-based screening, can efficiently propose novel molecular candidates with targeted electronic properties and favorable computed transport characteristics. The framework is intentionally modular: the generative model, the screening filters, and the MD validation can each be independently improved.

## Supplementary Material



## Data Availability

The generated molecular data set is deposited in a publicly accessible repository, please refer to the doi: 10.6084/m9.figshare.32070408

## Notes

Detailed description of the G-SchNet architecture, training protocols, and specific DFT/MD simulation parameters and functional group screening criteria (PDF)
