# Supplementary material for: Accelerating Electrolyte Discovery Using Generative Models and Molecular Dynamics Simulations
Source: ACS Omega. 2026 Aug 10;11(33):49205–13. doi: 10.1021/acsomega.6c01811 (PMC13520033; doi:10.1021/acsomega.6c01811)
Supplement: Supplementary file 1 [file ao6c01811_si_001.pdf]

Supporting Information

Accelerating Electrolyte Discovery Using Generative Models and Molecular Dynamics Simulations

Varisara Deerattrakul[a,b], *Salatan Duangdangchote*[c], *Adisak Boonchun*[d] *and Pawin Iamprasertkun*[a,b,*]

[a]School of Bio-Chemical Engineering and Technology, Sirindhron International Institute of Technology, Thammasat University, Pathum Thani 12120, Thailand

[b]Research Unit in Sustainable Electrochemical Intelligent, Thammasat University, Pathum Thani 12120, Thailand

[c]Maple Synapse Inc; North York M2K 0C4, Ontario, Canada

[d]Department of Physics, Faculty of Science, Kasetsart University, Bangkok 10900, Thailand

*Corresponding author. E-mail address: pawin@siit.tu.ac.th (P. Iamprasertkun).

Machine Learning Training and Screening

The inverse design of novel electrolyte solvents was conducted using G-SchNet[1] with SchNetPack 2.0[2], a conditional generative deep learning model. The model was trained on the QM9- QCDGE dataset[3] using a split ratio of 70:20:10 for training, validation, and testing, respectively, with a training batch size of 100. Following training, the model was conditioned on three sets of target quantum mechanical properties (Groups I, II, and III) to generate 5,000 molecular candidates per group. To ensure chemical and physical viability, all generated structures underwent a multi-stage validation filter: (1) 3D/Compositional Filter: Verification of valid 3D geometries and elemental consistency (H, C, N, O, F), (2) Structural Integrity: Selection of candidates that remained intact post-generation, resulting in 3,784 candidates for Group I, 3,573 for Group II, and 3,737 for Group III. Functional group screening protocol were applied to remaining molecules to exclude reactive or unstable motifs, including protic groups, nucleophiles, reactive carbonyls, and carboxylate esters. The filtered candidates were then subjected to high-fidelity DFT validation at the B3LYP/6-311++G(2d,2p) level to confirm their HOMO, LUMO, and dipole moment values. Subsequently, for the candidates selected for detailed analysis (A–F) and the conventional reference solvents, a broader DFT descriptor set is computed at the same level of theory: vertical ionization potential (vIP) and vertical electron affinity (vEA). [4]

Stokes−Einstein relation and mean square displacement (MSD) analysis

In molecular dynamic simulations, the self-diffusion coefficient (D) and transference number can be computed from the mean square displacement (MSD) analysis through the Stokes−Einstein relation:

$$D = \frac{1}{6} \times \lim_{t \to \infty} \left(\frac{\langle |\vec{r}(t) - \vec{r}(0)|^2 \rangle}{t} \right)$$

where D is the self-diffusion coefficient of specific ions, $\vec{r}(t)$ is the location of the center of mass of a specific ion at time t. The brackets indicate the ensemble average.

Whilst D of Li^+ and PF_6^- ions can be used to compute the Li^+ and PF_6^- transference number from these following equations:

$$t_{Li^+} = \frac{D_{Li^+}}{D_{Li^+} + D_{PF_6^-}}$$

$$t_{PF_6^-} = 1 - t_{Li^+}$$

where t_{Li^+} and $t_{PF_6^-}$ is the Li^+ and PF_6^- transference number, respectively.

Radial pair distribution function (RPDF) analysis

The structure analysis from the MD simulations can be described with the radial pair distribution function *g(r)*, which quantifies correlation between atom pairs *i* and *j*:

$$g_{ij}(r) = \frac{1}{4\pi r^2}\frac{1}{N\rho}\sum_{i=1}^{N}\sum_{j\neq i}^{N}\langle\delta(|r_{ij}| - r)\rangle$$

The coordination number *n(R)* over the specific distance *R* of specific ions can be obtained via this following integral:

$$n(R) = 4\pi\rho_j\int_0^R r^2 g_{ij}(r)dr$$

Where *r* is the location of the center of mass of a specific ion.

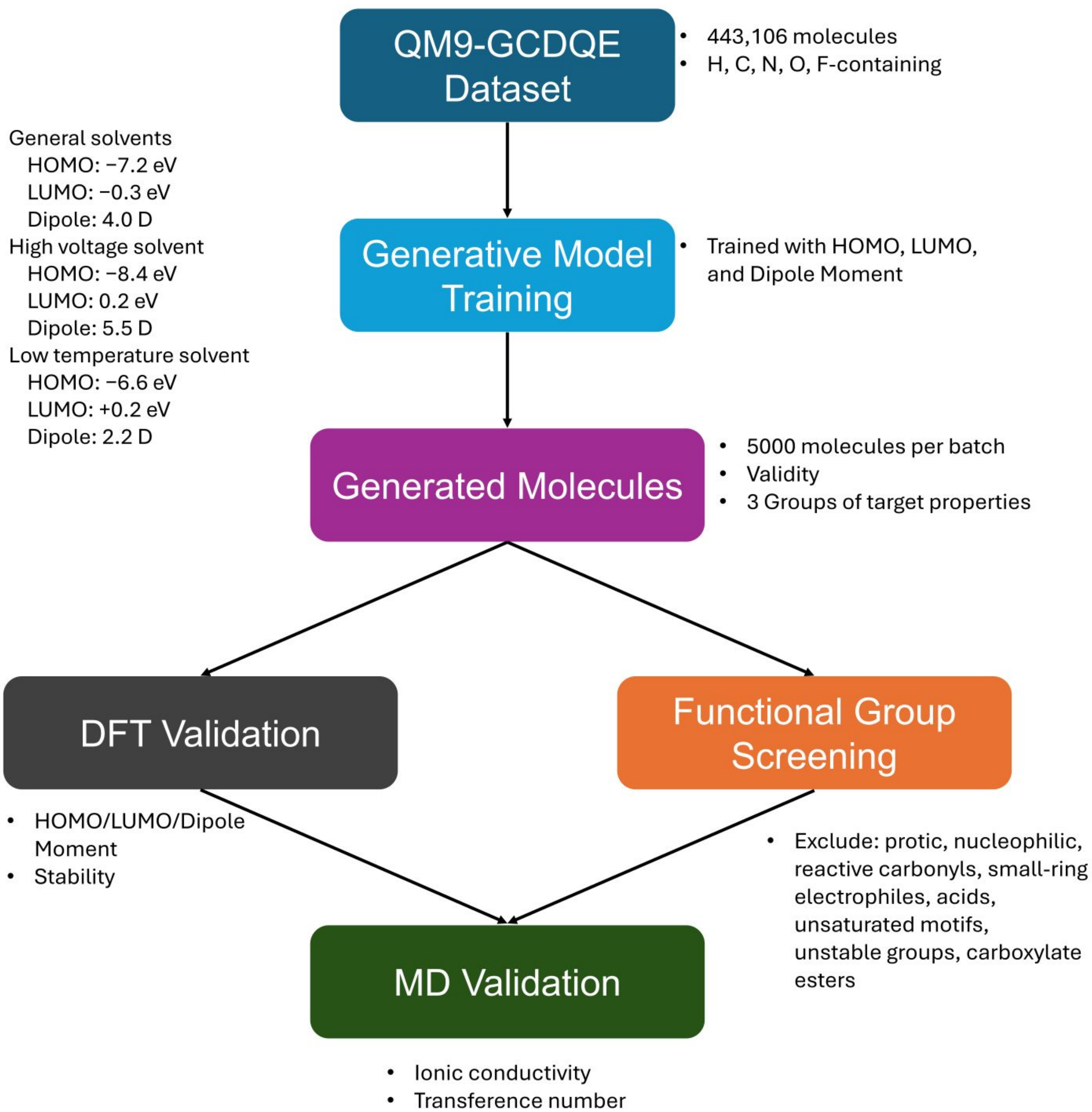

Figure S1. Computational workflow for the inverse design and validation of electrolyte solvents. The multi-stage pipeline illustrates the training of G-SchNet on the QM9- QCDGE dataset using target quantum mechanical properties (HOMO, LUMO, Dipole), followed by property-based molecular generation, a functional group screening, validation through DFT and Molecular Dynamics (MD) simulations.

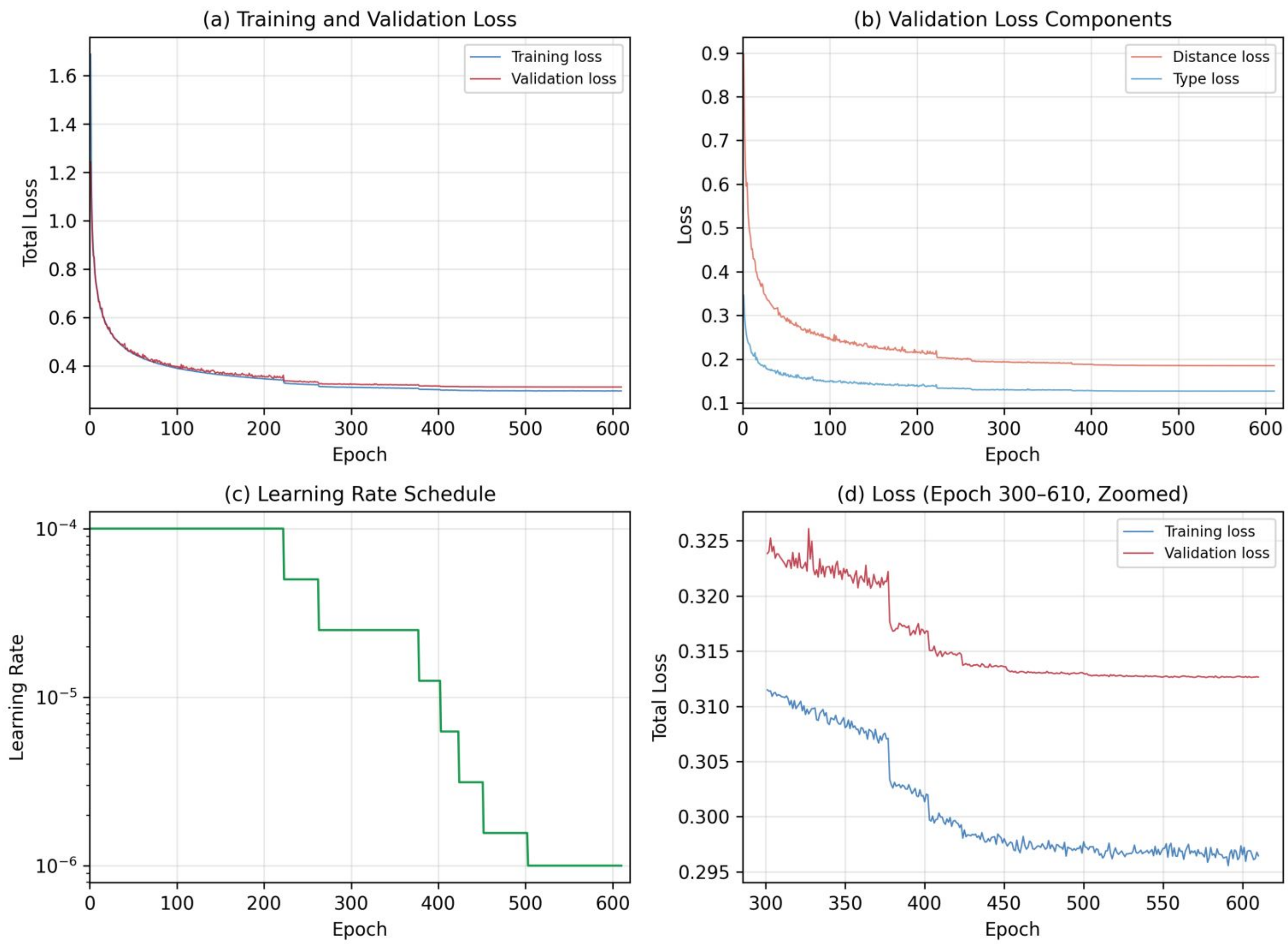

Figure S2. (a) Training and validation total loss. (b) Validation loss components. (c) LR schedule. (d) Total loss at the late stage of the training.

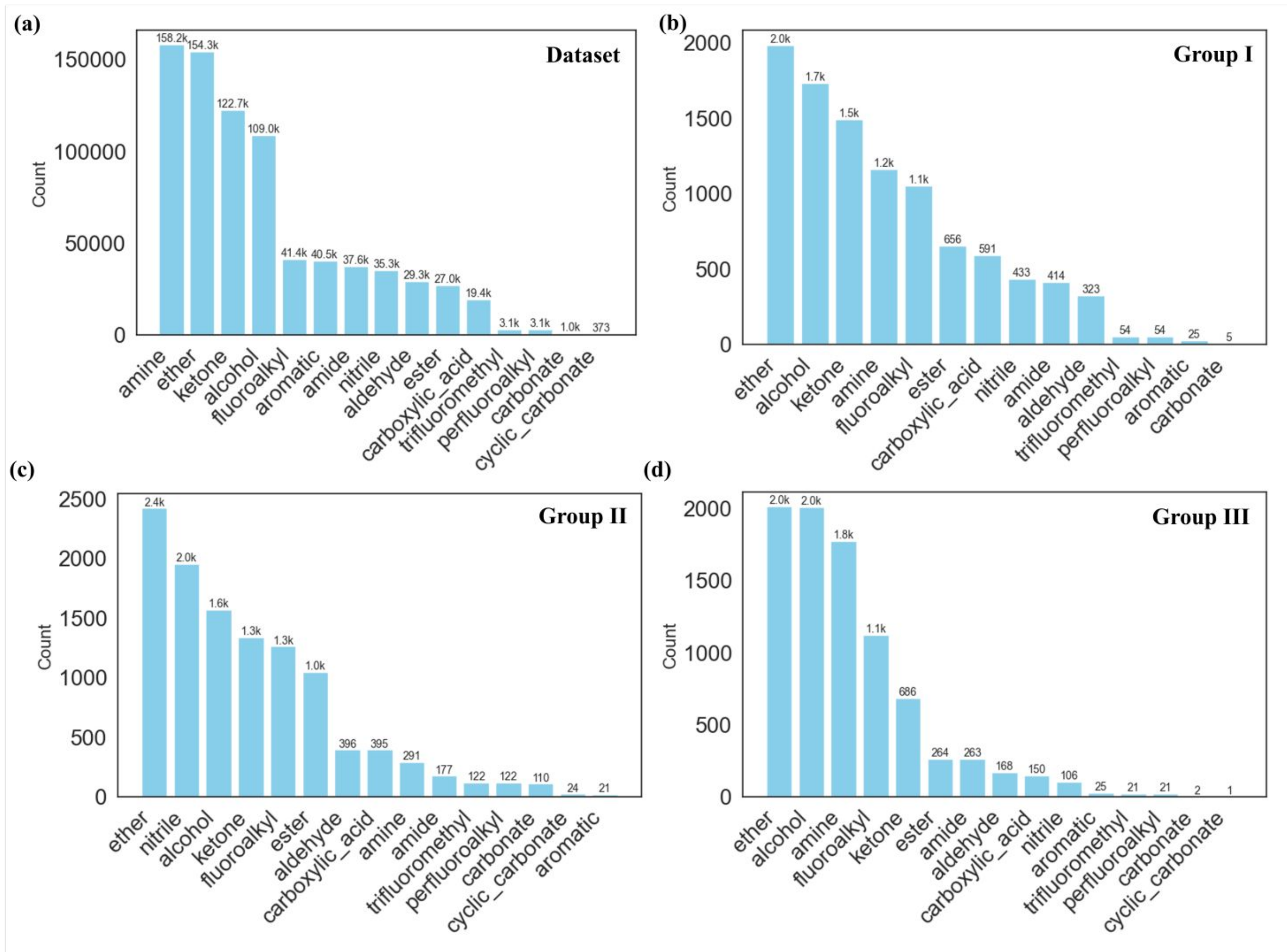

Figure S3. Functional group distribution in the training dataset and generated results. (a) Prevalence of functional groups in the original QM9- QCDGE dataset. (b-d) Distributions of functional groups within the generated candidates for Groups I, II, and III, highlighting the model's ability to maintain chemical diversity while focusing on target-specific motifs.

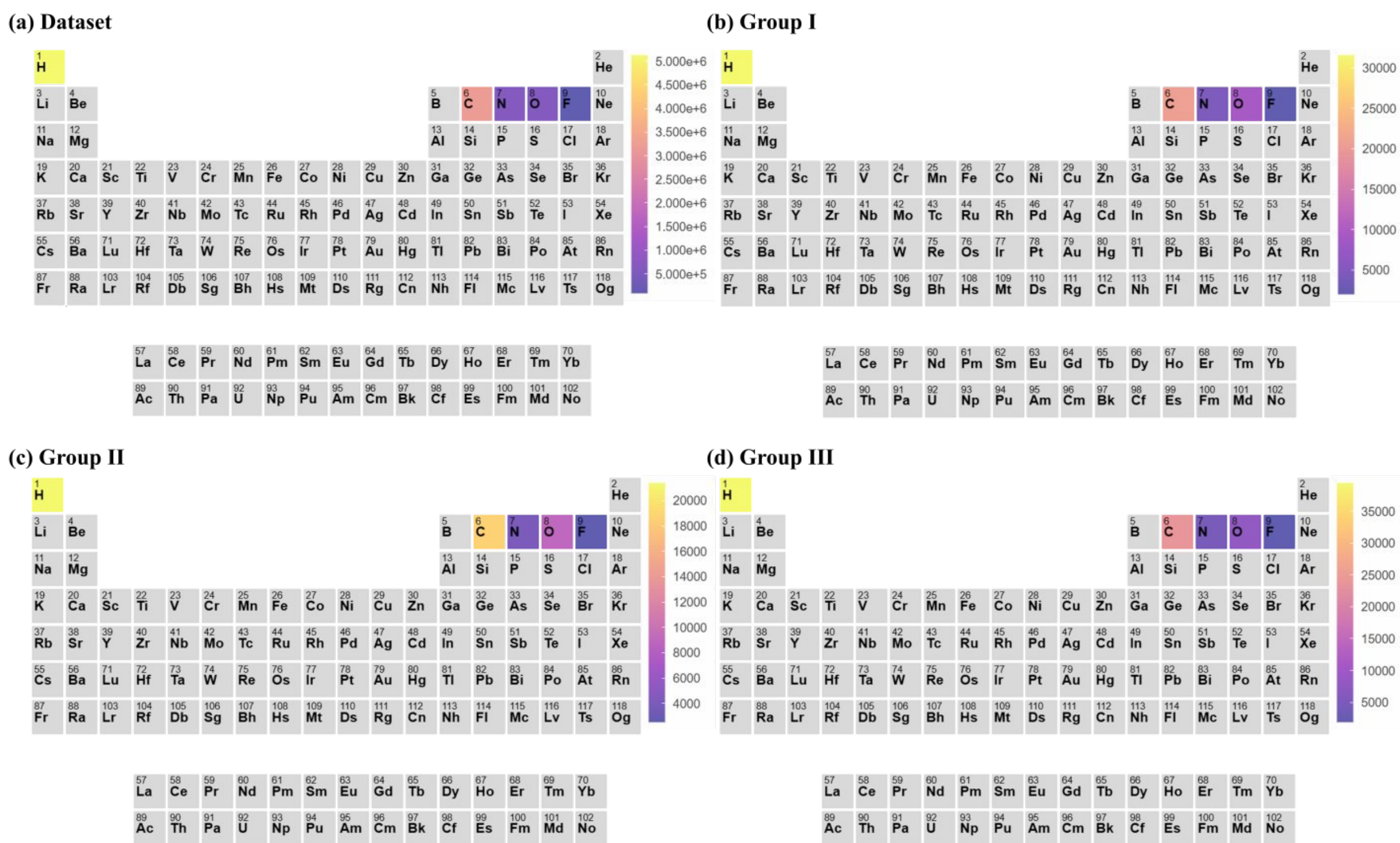

Figure S4. Elemental composition of the dataset and generated groups. Periodic table heatmaps showing the count of atoms (H, C, N, O, F) across (a) the training dataset and (b-d) the generated groups, confirming that the model effectively explored the chemical space defined by the input library.

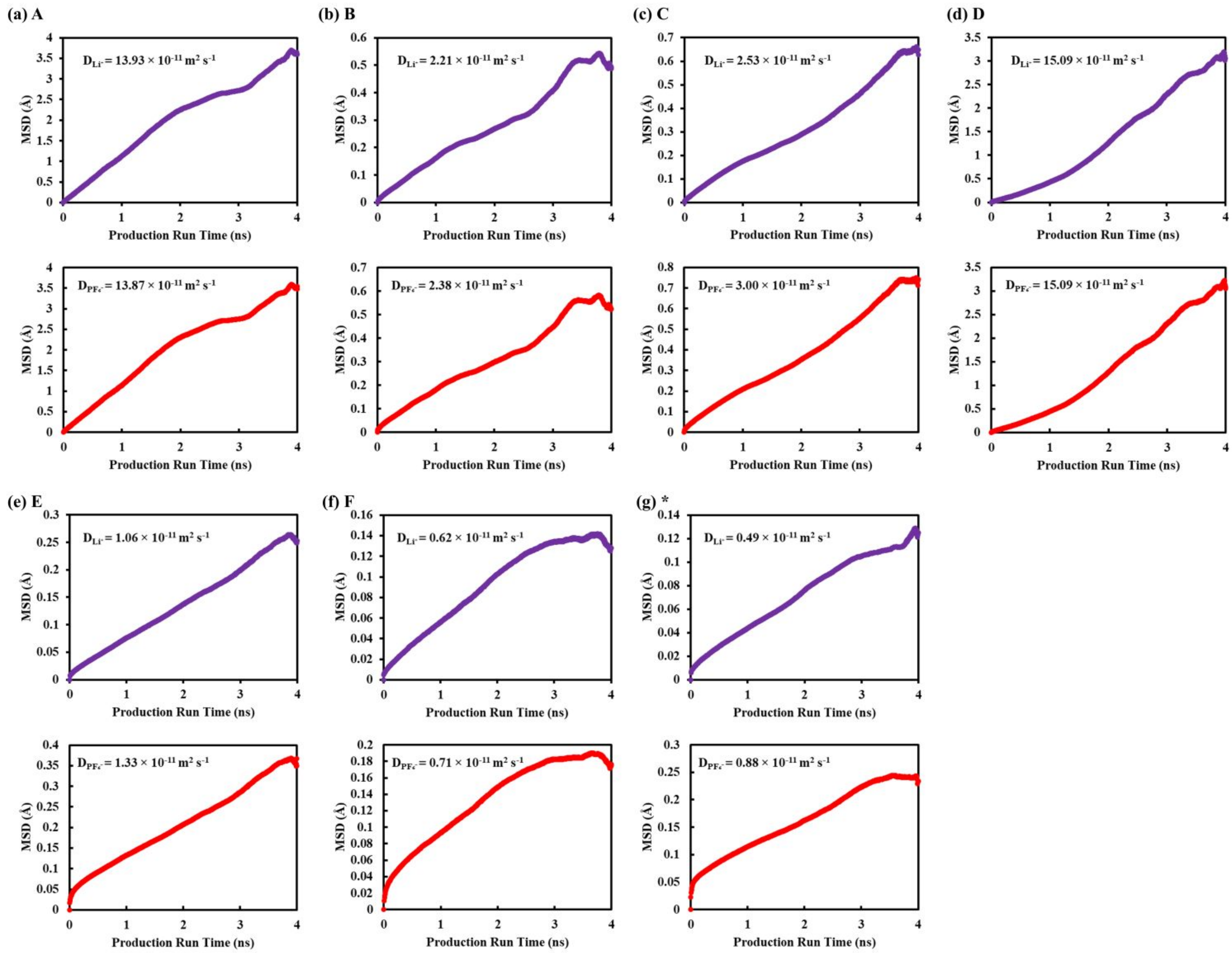

Figure S5. Mean Square Displacement (MSD) trajectories. Production run data for (a-f) candidates A–F and (g) the EC/DEC control. The linear region of the MSD for Li^+ (purple) and PF_6^- (red) was utilized to extract the self-diffusion coefficients (*D*) via the Einstein relation.

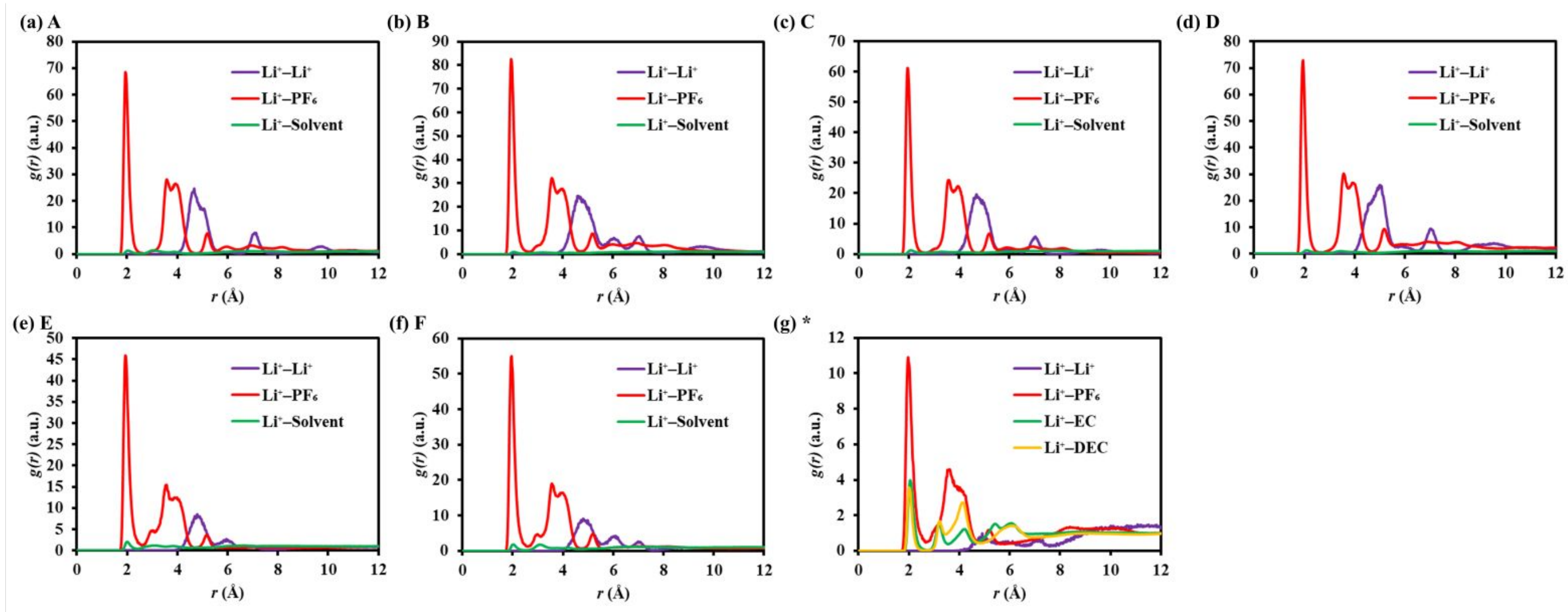

Figure S6. Radial pair distribution function *g(r)* of Li^+ with Li^+, PF_6^-, and solvents for (a) A, (b) B, (c) C, (d) D, (e) E, (f) F compounds, and (g) EC/DEC, respectively.

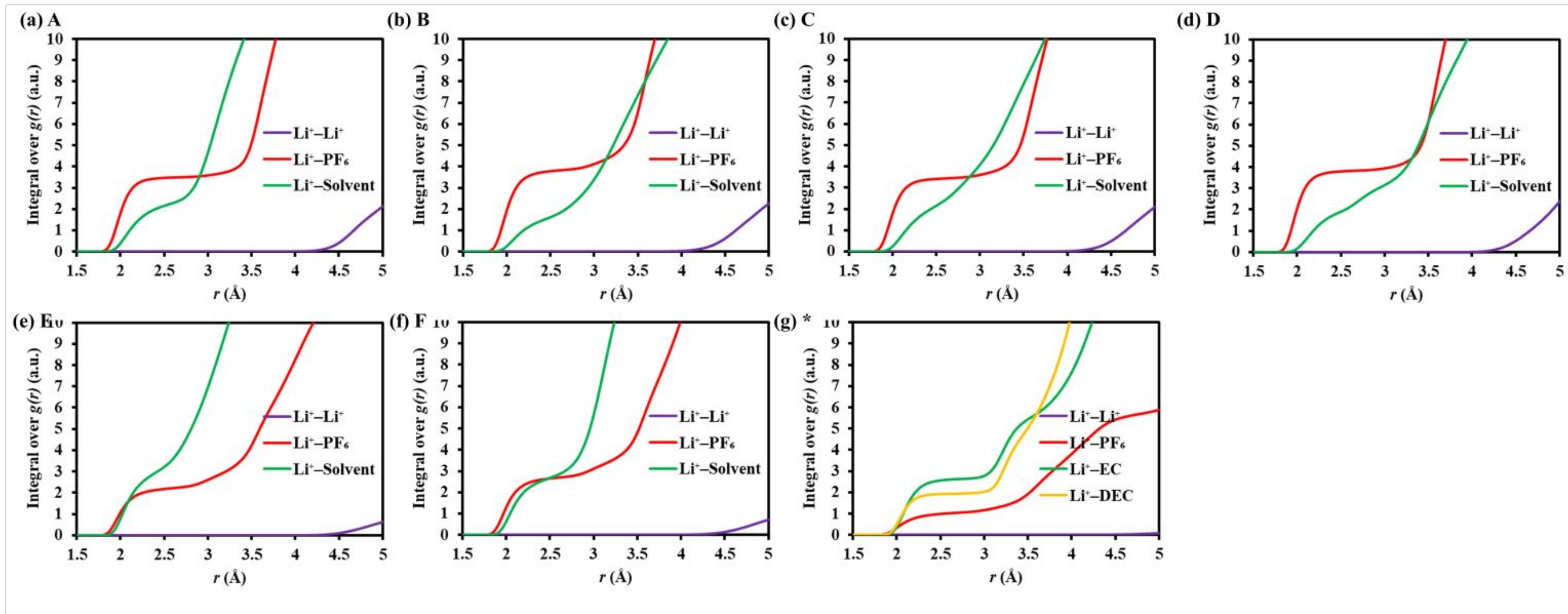

Figure S7. Integral over *g(r)* of Li^+ with Li^+, PF_6^-, and solvents for (a) A, (b) B, (c) C, (d) D, (e) E, (f) F compounds, and (g) EC/DEC, respectively.

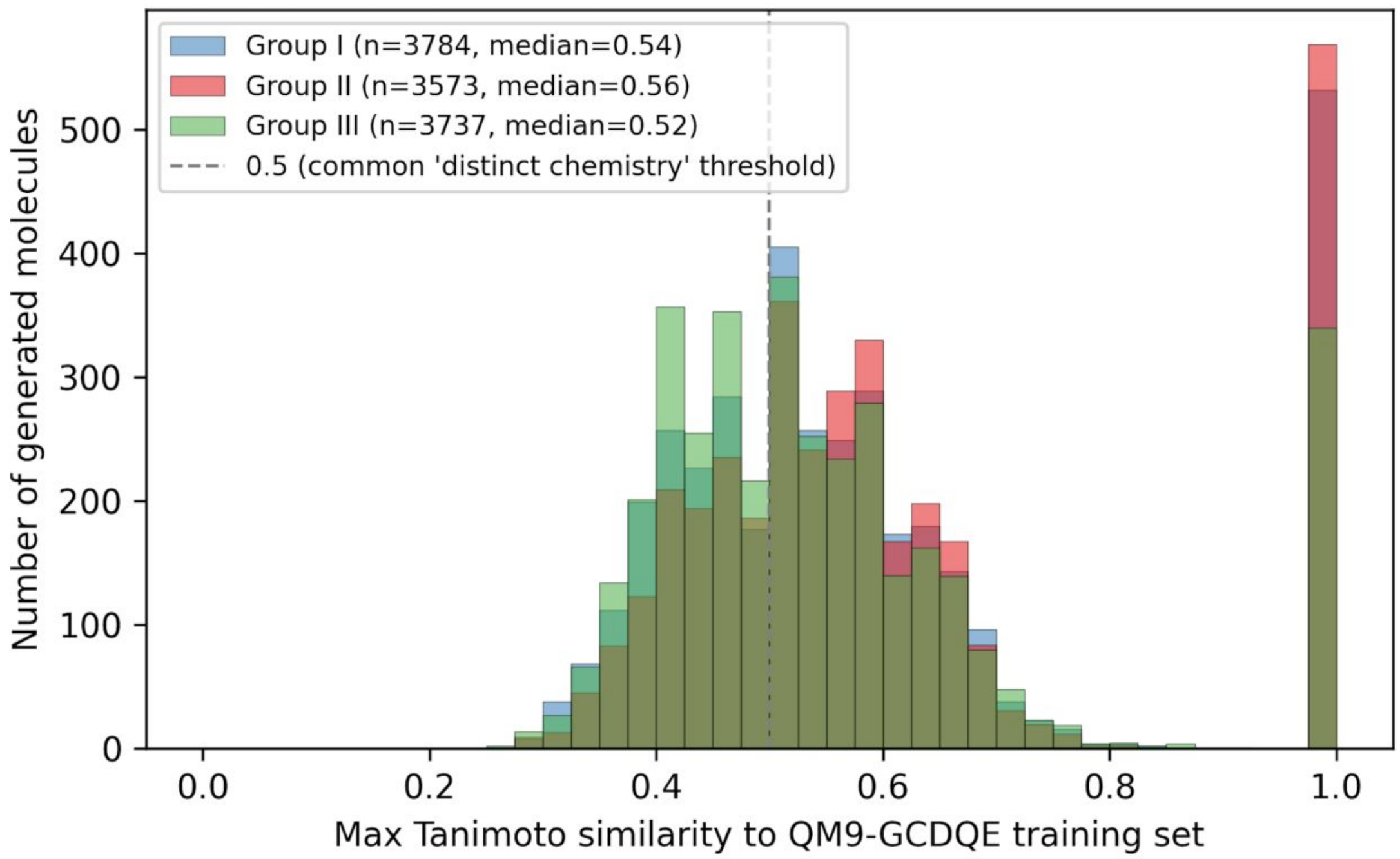

Figure S8. Tanimoto similarity distribution between generated molecules and their nearest neighbors in the QM9-GCDQE training set. Separate histograms are shown for Groups I (blue), II (red), and III (green).

Table S1. Initial configuration of the simulation cells

System		Li^+	PF_6^-	Solvents	EC	DEC
**A**	**No. of Molecule**	70	70	600	–	–
**B**	**No. of Molecule**	70	70	600	–	–
**C**	**No. of Molecule**	70	70	600	–	–
**D**	**No. of Molecule**	70	70	600	–	–
**E**	**No. of Molecule**	70	70	600	–	–
**F**	**No. of Molecule**	70	70	600	–	–
*** (EC/DEC)**	**No. of Molecule**	70	70	–	387	213

Table S2. Quantitative validation of the generative model. Validity, uniqueness, and novelty rates per target group, together with the mean absolute error (MAE) between target and DFT-computed property values across all valid generations. N refers to the post-screening pool that underwent DFT validation.

**Group**	N (generated)	N (valid)	Validity (%)	Uniquneness (%)	Novelty (%)	MAE HOMO (eV)	MAE LUMO (eV)	MAE μ (D)
I	5000	3784	75.7	100.0	91.1	0.485	2.337	1.305
II	5000	3573	71.5	100.0	90.1	0.834	2.757	2.032
III	5000	3737	74.7	100.0	94.7	0.466	1.875	1.051

Supplementary References.

(1) Gebauer, N. W. A.; Gastegger, M.; Hessmann, S. S. P.; Müller, K.-R.; Schütt, K. T. Inverse design of 3d molecular structures with conditional generative neural networks. *Nature Communications* **2022**, *13* (1), 973. DOI: 10.1038/s41467-022-28526-y.
(2) Schütt, K. T.; Hessmann, S. S. P.; Gebauer, N. W. A.; Lederer, J.; Gastegger, M. SchNetPack 2.0: A neural network toolbox for atomistic machine learning. *The Journal of Chemical Physics* **2023**, *158* (14). DOI: 10.1063/5.0138367 (acccessed 1/30/2026).
(3) Zhu, Y.; Li, M.; Xu, C.; Lan, Z. Quantum Chemistry Dataset with Ground- and Excited-state Properties of 450 Kilo Molecules. *Scientific Data* **2024**, *11* (1), 948. DOI: 10.1038/s41597-024-03788-x.
(4) Mester, D.; Kállay, M. Vertical Ionization Potentials and Electron Affinities at the Double-Hybrid Density Functional Level. *Journal of Chemical Theory and Computation* **2023**, *19* (13), 3982-3995. DOI: 10.1021/acs.jctc.3c00363.
